# Breathing life into learning about air quality: developing and implementing environmental health outreach with high school students

**DOI:** 10.5195/jmla.2025.1895

**Published:** 2025-07-01

**Authors:** Katie Hoskins, Nguyen Dang, Fahad Molla

**Affiliations:** 1 khoskins@touro.edu, Health Sciences Librarian and Assistant Professor, Touro University Nevada, Henderson, NV; 2 ngtdang@ucdavis.edu, California Health Sciences University, Clovis, CA; 3 molla2600@chsu.edu, California Health Sciences University, Clovis, CA

**Keywords:** Community Outreach, Instruction, High Schoolers, Environmental Health

## Abstract

**Background:**

The San Joaquin Valley (SJV) is comprised of diverse populations that cumulatively are medically underserved and experience significant air pollution. The SJV regularly has poor air quality and does not meet the state and federal attainment standards for particulate matter (PM) 2.5, ozone (eight hours), and PM 10. Air pollutants contribute to a high incidence of emergency room visits and hospitalizations for conditions like asthma. Because air pollutants correlate with built environments, these outcomes are concentrated geographically, which is a major driver of social determinants of health. Librarians from an osteopathic medical school in the SJV developed an educational outreach session to inform high school students about the causes and health impacts of air pollution and how they can find resources to educate themselves and others in their communities.

**Case Presentation:**

Two-hour interactive outreach sessions were provided at three local high schools in the SJV to students in health careers pathways. Librarians and student doctors guided high school students in health professions pathways through activities to understand the causes of poor air quality in the SJV; describe the different parts of the respiratory system and how air pollutants impact it; identify strategies for monitoring air quality and protecting their respiratory health; explore correlations between zip code and health outcomes; and access National Library of Medicine resources for reliable health information.

**Conclusion:**

Librarians can lead effective health outreach programming. High school students who participated in the outreach sessions increased their understanding of AQI, the causes of poor air quality, and the health effects of air pollution

## BACKGROUND

Over the years, the National Library of Medicine (NLM) has expanded its outreach efforts in support of its goal to facilitate access to high-quality health information [1]. Some of this work is done under the aegis of the National Network of Libraries of Medicine (NNLM), which provides funding for “projects that improve access to health information, increase engagement with research and data, expand professional knowledge, and support outreach that promotes awareness and use of NLM resources in local communities” [2].

In August 2021, NNLM Region 5, which includes California, announced a call for proposals for a $5,000 Environmental Health Outreach Award. Successful projects would focus on reaching underserved populations to increase awareness of environmental impacts on health or address a specific environmental health issue within underserved communities [3]. The lead author developed an educational outreach series funded by the NNLM to teach high school students in the San Joaquin Valley about the impact of poor air quality on the respiratory system, which is further described in the case presentation.

California's San Joaquin Valley (SJV) is home to an estimated 4.3 million people as of July 2021. Of these 4.3 million residents, 56% identify as Hispanic or Latino; however, some research suggests that the area's total population may be underestimated, especially among communities of color [4]. The average median household income in the SJV is approximately $63,708 compared to the state median household income of $84,097 [4]. The SJV is also a medically underserved area, with the second lowest ratio of primary care physicians (47 per 100,000 population in California) and the lowest ratio of specialists (81 per 100,000 population in California) [5].

The SJV regularly has poor air quality and does not meet the state and federal attainment standards for particulate matter (PM) 2.5 and ozone (eight hours), nor does it meet the state attainment standards for PM 10 [6]. Air pollutants contribute to a high incidence of emergency room visits and hospitalizations for conditions like asthma [7, 8]. Because air pollutants correlate with built environments that themselves reflect systemic inequities like historical redlining practices, these negative health outcomes are concentrated geographically, which is a major driver of social determinants of health [9, 10].

## CASE PRESENTATION

### Goals and Learning Activities

The project leaders decided to provide in-person sessions for high schools from rural and underserved areas in the Fresno and Madera regions. The goals were for high school students in health professions pathways to: understand the causes of poor air quality in the SJV; describe the different parts of the respiratory system and how air pollutants impact it; identify steps they can take to monitor the air quality and adjust their activities to protect their health; and access NLM resources for health information. Based on these goals and the class duration of two hours, activities were organized into five stations, which students rotated through approximately every 20 minutes.

Station 1: Respiratory System. Medical students used an anatomical model to demonstrate the air pathways as well as the functions of other parts of the respiratory system like alveoli, before offering one or more of the following interactive activities. High school students were asked to take a deep breath and hold it, counting how long they were able to hold their breath. Then they were asked to hyperventilate (breathe very quickly) for 5 seconds and hold their breath, counting how long they were able to hold it. This demonstrated that the amount of time they could hold their breath increased after hyperventilation due to how the respiratory system works. Another activity had students do jumping jacks while breathing through a straw to simulate breathing with asthma.

Station 2: Air Pollution and the Respiratory System. Medical students played clips from three videos and discussed the concepts with high school students. The first video was “The Air We Breathe-Inside Your Lungs,” which discussed the type of air pollutants (PM 2.5 and PM 10) and how they affect the lungs. The California Health Sciences University Simulation Center produced a second video, which showed a patient in respiratory distress. The medical student periodically paused the video to ask students questions and provide background information. A 1.5-minute clip of a third video, “What is Asthma? - Pathophysiology of Asthma,” explained how short and long-term inhalers relieve asthma symptoms.

Station 3: Types of Pollutants, AQI, and Outdoor Activities. Faculty librarians took high school students on a walk outside the building to measure Air Quality Index (AQI) using AirBeams and mobile phones that were distributed to small groups of students [11]. After making an initial measurement, the librarians explained the definition of AQI and different particle sizes (PM 2.5 and PM 10). Students were asked questions like “What do you think gives off the most pollution around or inside the school?” “Around or inside your home?,” and “Why is it important to identify these causes?” The students were then instructed to measure AQI at different heights and distances apart to compare the differences in values. After the walk, the librarians compiled a graph of measured values and discussed with the students the change in values throughout the walk. In the end, students were shown a video on how AQI value changes when devices are close to car exhaust.

Station 4: National Library of Medicine (NLM) and Community Resources. A faculty librarian demonstrated how to find reliable health information on the websites for MedlinePlus and TeensHealth through a combination of print handouts and online quiz questions [12, 13]. The librarian also shared local online resources to help high school students discover the current AQI and implications for outdoor physical activity, which included Healthy Air Living Schools Program, Real-Time Air Advisory Network (RAAN), San Joaquin Valley Air District, and AirNow [14-17].

Station 5: Causes of Air Pollution and Health Impact Disparities. A faculty librarian provided an overview of the causes of poor air quality in the SJV, the various bodily systems impacted by poor air quality, and why zip code is a fairly accurate predictor of health. Students were asked what they thought were the three most common causes of poor air quality before being given the opportunity to compare their responses with the known causes. Another activity involved the use of the California Office of Environmental Health Hazard Assessment (OEHHA)'s CalEnviroScreen 4.0, which can be used to help identify California communities that disproportionately experience pollution [9]. Students were able to see that the SJV has a higher pollution burden and population sensitivities than other regions in California [9], as well as variations within the SJV itself. The last activity involved the students entering their zip codes onto the Healthy Fresno County Community Dashboard to see the rates of health conditions like asthma for that zip code and how it compared to county and state levels [18]

## RESULTS

Pre- and post-surveys were implemented to assess student learning. There were a total of 93 responses for the pre-survey. Of the 57 students who completed the post-survey, the last four digits of their phone numbers were matched to the pre-survey, leaving a total of 50 post-survey responses to compare with the pre-survey.

In the pre-survey, high school students were asked 11 questions about AQI and its health impacts (see supplement/appendix). In Question 4 (Q4), students were asked to describe their understanding of AQI and how to modify their daily activities accordingly. A rubric was used to evaluate responses. Students got 2 points for knowing the full definition of AQI and then 2 points for mentioning changes to outdoor activity, with partial points given. Table 1 summarizes the results, which showed that after the educational outreach activities, the students had a statistically significant increase in understanding of AQI definition and overall understanding of AQI. There was no statistically significant difference regarding modification of daily activities.

**Table 1:** AQI Knowledge as measured by Q4, in which students were asked the definition of AQI and what daily activities should be changed to accommodate for poor air quality.

		AQI Definition	Daily Activities
**Mean (Pre)**		0.48	0.52
**Mean (Post)**		0.98	0.66
**Mean Difference**		-0.50	-0.14
**Standard Deviation**		1.02	0.89
**Standard Error of Mean**		0.14	0.13
**95% CI**	**Lower**	-0.78	-0.39
	**Upper**	-0.22	0.11
**t-value (paired t-test)**		-3.49	-1.10
**Degree of Freedom**		49	49
**p-value**		0.00064	0.14

Question 6 asked students to identify which body systems were impacted by air pollutants: respiratory system only; respiratory and circulatory systems; respiratory, circulatory, and neurological systems; or respiratory, circulatory, and neurological systems and premature death. In pre-survey Q6, most students thought that the air quality only impacted the respiratory system (38%) or both the respiratory and circulatory systems (30%), which is incorrect. Only 20% of students correctly identified that air quality impacts the respiratory, circulatory, and neurological systems as well as premature death. Post-survey, 76% of students selected the correct answer, which is a statistically significant increase, t(49) = -9.92, p < 0.001

**Figure 1:**
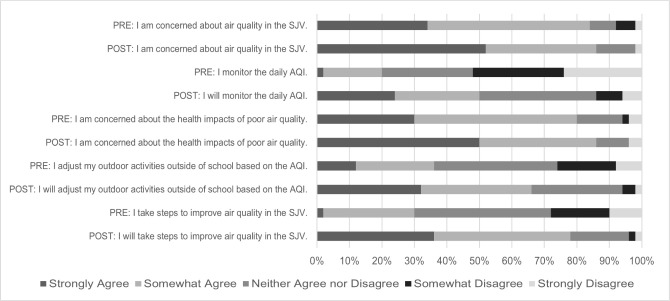
Comparison of Pre and Post Survey Responses for Q7

Question 7 consisted of a five-point Likert scale assessing students' concern about air quality in the SJV, their concern about the health impacts of air pollution, whether they did/will monitor the daily AQI, whether they did/will adjust their outdoor activities based on AQI, and whether they did/will take steps to improve air quality. Student responses to this question are visualized in Figure 1.

## DISCUSSION

From the responses collected, the high school students who participated in the outreach sessions increased their understanding of AQI, the causes of poor air quality, and the health effects of air pollution. Prior to the intervention, students' knowledge of AQI (Q4) was poor overall with an average pre-survey score of 0.48 out of 2.0. Students also did not understand how the AQI value impacts recommendations for engaging in daily activities outdoors (Q4) as seen with the pre-survey score of 0.52 out of 2.0. Following the intervention, students' performance on the post-test demonstrated statistically significant improvement in their understanding of AQI. On the other hand, the data do not show a significant difference in students' understanding of the impacts of AQI on daily activities after the intervention. It is unclear whether these differences in the impact of the intervention on student learning reflect the content covered (e.g., perhaps there was insufficient discussion of how to modify outdoors activities based on AQI), or if this reflects that it is harder to change people's behaviors than to increase their knowledge.

In pre-survey Q6, most students thought that the air quality only impacted the respiratory system (38%) or both the respiratory and circulatory systems (30%), which is incorrect. Only 20% of students correctly identified that air quality impacts the respiratory, circulatory, and neurological systems as well as premature death. Post-survey, 76% of students selected the correct answer. This suggests that the health outreach session accomplished its goal of improving students' understanding of the wide-ranging health impacts of poor air quality.

One interesting tension in our findings is that while most students (84%) in the pre-survey indicated their concern about air quality in the SJV and understood some of the health impacts of poor air quality (Q7), their prior knowledge of AQI was limited (Q4) and that few students reported adjusting their outdoor activities to reflect air quality (Q7). This may indicate a disconnect between students' perception of the air quality of their communities and their understanding of how air quality is empirically measured. Reviewing the pre and post survey responses for Q7 about monitoring the daily AQI, the biggest difference occurred in those who disagreed about monitoring AQI, declining from 52% to 14%. It appears that the activity in which students took a walk and measured the AQI using AirBeams may have influenced their understanding of the value of monitoring AQI. This finding aligns with prior work in the area, which has found that hands-on health outreach programming can lead to meaningful community-based learning opportunities [19-22].

Limitations of these outreach sessions include that there was only a single two-hour session and no long-term follow-up. Similar future outreach efforts could consider multiple sessions, which would enable additional interactive activities and more in-depth discussion. Following up with students several months after the learning activities could examine long-term learning retention, as well as if students were still making changes to reduce air pollution and/or mitigate the health impacts of poor air quality. Such a follow-up would have been a valuable comparison with the results of Dorevitch et al.'s study, which had participants complete a follow-up questionnaire a year after the instructional intervention and found that many participants demonstrated limited recall of the knowledge gained twelve months prior [20].

## Data Availability

Data associated with this article are available in the Open Science Framework at https://osf.io/btwqa/.
